# Limits of Calcium Clearance by Plasma Membrane Calcium ATPase in Olfactory Cilia

**DOI:** 10.1371/journal.pone.0005266

**Published:** 2009-04-23

**Authors:** Steven J. Kleene

**Affiliations:** Department of Cancer and Cell Biology, University of Cincinnati, Cincinnati, Ohio, United States of America; University of Oldenburg, Germany

## Abstract

**Background:**

In any fine sensory organelle, a small influx of Ca^2+^ can quickly elevate cytoplasmic Ca^2+^. Mechanisms must exist to clear the ciliary Ca^2+^ before it reaches toxic levels. One such organelle has been well studied: the vertebrate olfactory cilium. Recent studies have suggested that clearance from the olfactory cilium is mediated in part by plasma membrane Ca^2+^-ATPase (PMCA).

**Principal Findings:**

In the present study, electrophysiological assays were devised to monitor cytoplasmic free Ca^2+^ in single frog olfactory cilia. Ca^2+^ was allowed to enter isolated cilia, either through the detached end or through membrane channels. Intraciliary Ca^2+^ was monitored via the activity of ciliary Ca^2+^-gated Cl^−^ channels, which are sensitive to free Ca^2+^ from about 2 to 10 µM. No significant effect of MgATP on intraciliary free Ca^2+^ could be found. Carboxyeosin, which has been used to inhibit PMCA, was found to substantially increase a ciliary transduction current activated by cyclic AMP. This increase was ATP-independent.

**Conclusions:**

Alternative explanations are suggested for two previous experiments taken to support a role for PMCA in ciliary Ca^2+^ clearance. It is concluded that PMCA in the cilium plays a very limited role in clearing the micromolar levels of intraciliary Ca^2+^ produced during the odor response.

## Introduction

Calcium ion is widely used as in intracellular messenger. Many signal-transduction processes cause cytoplasmic Ca^2+^ to increase. However, Ca^2+^ becomes toxic at high levels, and several mechanisms are known that limit or reverse increases in cytoplasmic Ca^2+^ [1]. In theory, Ca^2+^ clearance can be an acute problem in any dendrite, cilium, or other cellular process with an enormous surface-to-volume ratio. In such a process, a small influx of extracellular Ca^2+^ could quickly elevate cytoplasmic Ca^2+^ to a toxic concentration. How such fine cellular compartments clear Ca^2+^ is not well understood.

The vertebrate olfactory cilium is an attractive system for studying Ca^2+^ clearance in fine organelles. Olfactory signal transduction occurs on these cilia, which extend from the tip of each olfactory receptor neuron into the mucus [2]. It is known that Ca^2+^ accumulates within the cilium during odor transduction. Transduction begins when an odorant molecule binds to a receptor on the membrane of a cilium. A G-protein-coupled cascade produces cyclic AMP (cAMP), which leads to a depolarization caused by a sequence of two ciliary membrane channels [reviewed in ref. 3]. cAMP gates cyclic-nucleotide-gated (CNG) channels, which allow a depolarizing influx of Ca^2+^ and Na^+^ [3], [4]. The Ca^2+^ then gates Cl^−^ channels, which generate a further inward current via an efflux of Cl^−^ [5]–[7]. Experimental estimates of intraciliary free Ca^2+^ during the odor response range from 300 nM [8] to 100 µM [9]. Thus mechanisms must exist to clear Ca^2+^ from the ciliary cytoplasm. However, there is little direct evidence that Ca^2+^ clearance is a function of the cilium itself.

Several studies suggest that the plasma membrane Ca^2+^-ATPase (PMCA, also called Ca^2+^ pump; [refs. 1], [10]) may contribute to Ca^2+^ clearance following the odor response [11]–[16]. By monitoring cytoplasmic Ca^2+^ in isolated olfactory cilia, I have found that PMCA in the cilium contributes little to clearance of Ca^2+^ at the micromolar levels seen during the odor response.

## Materials and Methods

Electrical recordings were made from olfactory cilia of Northern grass frogs (*Rana pipiens*) as described elsewhere [17]. Frog olfactory epithelium was dissociated by mechanical shredding. One cilium of an isolated olfactory receptor neuron was drawn into a patch pipette, and a high-resistance seal was made where the olfactory knob meets the base of the cilium. The cilium was then excised from the cell, resulting in an inside-out patch configuration. The pipette containing the cilium was moved through the air to various cytoplasmic baths; in each the intracellular side of the cilium was exposed to the bath solution. All procedures were approved by the Institutional Animal Care and Use Committee of the University of Cincinnati.

The extracellular (pipette) solution contained (in mM): NaCl 115; KCl 3; Na-HEPES 5; MgCl_2_ 2; CaCl_2_ 1; pH 7.2. The control cytoplasmic (bath) solution contained (in mM): NaCl 110; KCl 5; K_4_BAPTA 2; K-HEPES 5; MgCl_2_ 2; CaCl_2_ 1; pH 7.2. The concentration of free Ca^2+^ in this bath was 0.1 µM. To make bath solutions with 7 µM free Ca^2+^, BAPTA was replaced with 2 mM dibromoBAPTA and CaCl_2_ was increased to 1.7 mM. To make bath solutions with 20 µM free Ca^2+^, BAPTA was replaced with 2 mM HEDTA and CaCl_2_ was increased to 1.2 mM. To make the bath with 300 µM free Ca^2+^, the 2 mM BAPTA was saturated by adding 2.3 mM CaCl_2_. Other bath solutions contained additions of ATP, ITP, or cAMP as indicated. Concentrations of free Ca^2+^ were estimated by the method of Bers [18] as described previously [19].

In any given bath, the current-voltage relation usually stabilized within 15 s. This stability was verified before transferring the cilium to a new bath. Between tests in solutions expected to activate currents, the cilium was kept in an appropriate control bath for 2 min. Before testing for effects of ITP or ATP, the cilium was kept in a control bath containing the same nucleotide. Thus the nucleotide was already present in the cilium when the recordings commenced.

DibromoBAPTA was purchased from Molecular Probes/Invitrogen (Carlsbad, CA) and other reagents from Sigma-Aldrich (St. Louis, MO). 5(6)-carboxyeosin diacetate was prepared as a 5 mM stock solution in DMSO and diluted from that to a final concentration of 0.01 to 50 µM. The same final concentration of DMSO (≤1% *v/v*) was also included in all control baths in those studies.

For electrical recording, both the recording pipette and chamber were coupled to an Axopatch 200B patch-clamp amplifier by Ag/AgCl electrodes. All recordings were made under voltage-clamp at room temperature (25°C) using pCLAMP 5.5 data-acquisition software (Axon Instruments/Molecular Devices, Union City, CA). A small leak current measured in the control bath was subtracted when quantifying the recordings. This leak has not been subtracted from the recordings shown.

To measure the onset time of the Ca^2+^-activated Cl^−^ current, average current values during the initial plateau and final plateaus were calculated. The Ca^2+^-activated Cl^−^ current accounts for the current between these plateaus. The onset time was arbitrarily defined as the time at which 10% of the Cl^−^ current appeared. This was measured by interpolation after digitally smoothing the recording by averaging every 20 adjacent points.

Effects of reagents were tested by comparing the currents measured in one cilium in each of two baths (e.g. with or without ATP). The significance of any difference between the two treatments was calculated with Student's *t*-test for repeated measures. Significance was claimed only when *P*<0.05.

## Results

If PMCA is able to clear Ca^2+^ from the cilium, it should be possible to demonstrate an ATP-dependent decrease in intraciliary Ca^2+^. To follow changes in intraciliary Ca^2+^, two assays were developed that use the ciliary Ca^2+^-gated Cl^−^ channel as a sensor of free Ca^2+^. Its useful range as a sensor is about 2 to 10 µM [19]–[21]. This range is also typically sufficient to activate PMCA [1], [10], [13].

**Figure 1:**
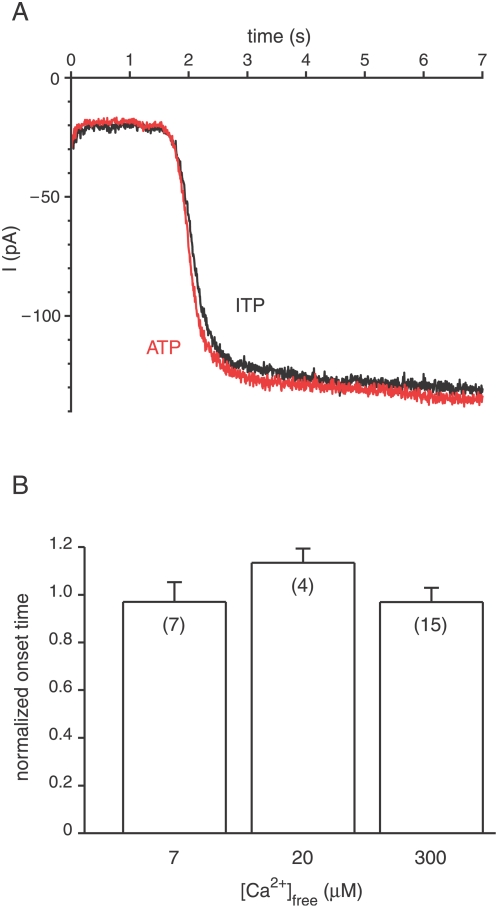
Cl^−^ current activated by longitudinal diffusion of Ca^2+^ from the bath is insensitive to ATP. (A) A cilium was held for 2 min in a control cytoplasmic bath containing 0.1 µM free Ca^2+^ and 2 mM MgITP. At *t* = 0 s, the cilium was transferred to a bath containing 300 µM free Ca^2+^ and 2 mM MgITP (recording shown in black). Membrane potential was held at −50 mV. The initial current (−20 pA) is a membrane leak current. The additional current that starts at *t* = 1.8 s is the Ca^2+^-activated Cl^−^ current. The experiment was repeated in control and test solutions in which MgITP was replaced by MgATP (recording shown in red). Ca^2+^ was buffered in all solutions with 2 mM BAPTA. (B) Summary of the effects of ATP on the onset time of the Cl^−^ current. Normalized onset time is the time measured in the presence of ATP divided by that measured in the control (ITP). The normalized onset times shown are 0.97±0.08 (7 µM free Ca^2+^), 1.13±0.06 (20 µM free Ca^2+^), and 0.97±0.06 (300 µM free Ca^2+^).

For the first assay, a pipette containing a cilium was held in a cytoplasmic bath containing 0.1 µM free Ca^2+^, allowing this solution to fill the cilium. The pipette containing the cilium was then transferred to a cytoplasmic bath with a high level of free Ca^2+^. Solutions on both sides of the ciliary membrane contained Cl^−^. As Ca^2+^ diffused into the cilium from the bath, some of the Ca^2+^ molecules gated Cl^−^ channels. Since the inside of the cilium was held more negative than the equilibrium potential for Cl^−^, an inward Cl^−^ current (i.e. a Cl^−^ efflux) resulted. Recordings from two such experiments are shown in Fig. 1A; note the inward (negative) Cl^−^ current that developed beginning at *t* = 1.8 s.

If ATP were able to support the extrusion of 2 to 10 µM Ca^2+^ from the cilium, one would expect to see a slower development of the Cl^−^ current in the presence of 2 mM cytoplasmic MgATP than in its absence. The onset time of the Cl^−^ current was measured in both the presence and absence of 2 mM MgATP as Ca^2+^ was allowed to diffuse into the cilium. There was no significant difference in the onset times (Fig. 1B; a normalized onset time of 1.0 indicates no effect). The amplitude of the Cl^−^ current also had no dependence on the presence of ATP (not shown). These conclusions held when baths containing any of three different concentrations of free Ca^2+^ were tested (Fig. 1B). In the control experiments, ITP replaced ATP. ITP is not a substrate for PMCA [22]–[24] but controls for a slight buffering of Ca^2+^ by nucleotides.

**Figure 2:**
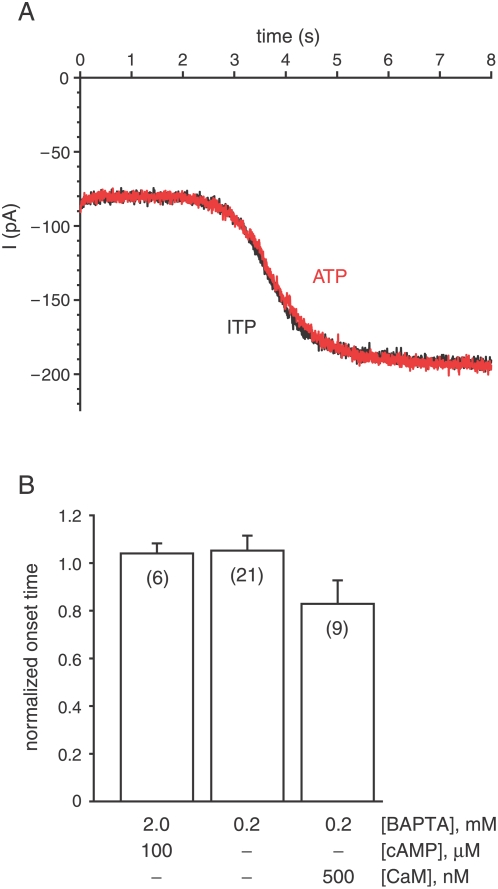
Cl^−^ current activated by membrane Ca^2+^ influx is insensitive to ATP. (A) A cilium was held at 0 mV for 2 min in a control cytoplasmic bath containing 0.1 µM free Ca^2+^, 100 µM cAMP, and 2 mM MgITP. Ca^2+^ was buffered with 2 mM BAPTA. At *t* = 0 s, the voltage was switched to −40 mV (recording shown in black). The initial current (−80 pA) is carried by cations, including Ca^2+^, primarily through CNG channels gated by cAMP. The additional current that starts at *t* = 2.9 s is the Ca^2+^-activated Cl^−^ current. The experiment was repeated in control and test solutions in which MgITP was replaced by MgATP (recording shown in red). (B) Summary of the effects of ATP on the onset time of the Cl^−^ current. Normalized onset time is as defined for Fig. 1. The normalized onset times shown are 1.04±0.04 (left bar), 1.05±0.06 (middle bar), and 0.82±0.10 (right bar).

A second assay more closely mimicked the natural activation of the Cl^−^ channels during the odor response. An excised cilium was placed in a cytoplasmic bath containing cAMP. The extracellular (pipette) solution contained 1 mM Ca^2+^, and both solutions contained Cl^−^. Initially, the voltage was clamped at the reversal potential for the cAMP-activated current (0 mV). So although the CNG channels were gated by the cAMP, there was almost no net current at the start of the experiment. When the voltage was jumped to a negative potential, cations including Ca^2+^ carried an inward current through the CNG channels (the initial steady current of −80 pA in each recording of Fig. 2A). As intraciliary Ca^2+^ accumulated, it became concentrated enough to gate the Cl^−^ channels, and a second increment of current gradually appeared (beginning at *t* = 2.9 s in Fig. 2A). It is known that this additional current is carried by Cl^−^ [6], [20]. In this second assay, there was again no significant difference in the onset times that depended on the presence of ATP (Fig. 2B, first bar).

The strong Ca^2+^ buffer used (2 mM BAPTA) might have competed with PMCA for Ca^2+^ and made detection of Ca^2+^ pumping more difficult. The test was repeated in baths with just 0.2 mM BAPTA. In this condition, addition of cAMP is unnecessary. Spontaneous gating of the CNG channels allows a Ca^2+^ influx sufficient to gate the Cl^−^ channels [6], [20]. Even with reduced Ca^2+^ buffering, the onset of the Cl^−^ current was not significantly changed by the addition of ATP (Fig. 2B, second bar). One cilium in this group did show a complete and reversible block of the secondary Cl^−^ current in the presence of ATP. However, no other cilium among the 21 tested was similar.

PMCA is effective at much lower Ca^2+^ levels in the presence of calmodulin (CaM) [1], [10], [13]. In vesicles enriched in olfactory cilia, CaM enhanced PMCA transport with a half-maximal concentration of 31 nM [13]. In single cilia, the presence of 500 nM CaM and 2 mM MgATP decreased the onset time of the Cl^−^ current (Fig. 2B, third bar), but this decrease was not significant. If ATP and CaM were to activate PMCA, the onset time would be expected to increase. In none of the studies shown in Fig. 2 was there an ATP-dependent change in the amplitude of the Cl^−^ current (not shown).

**Figure 3:**
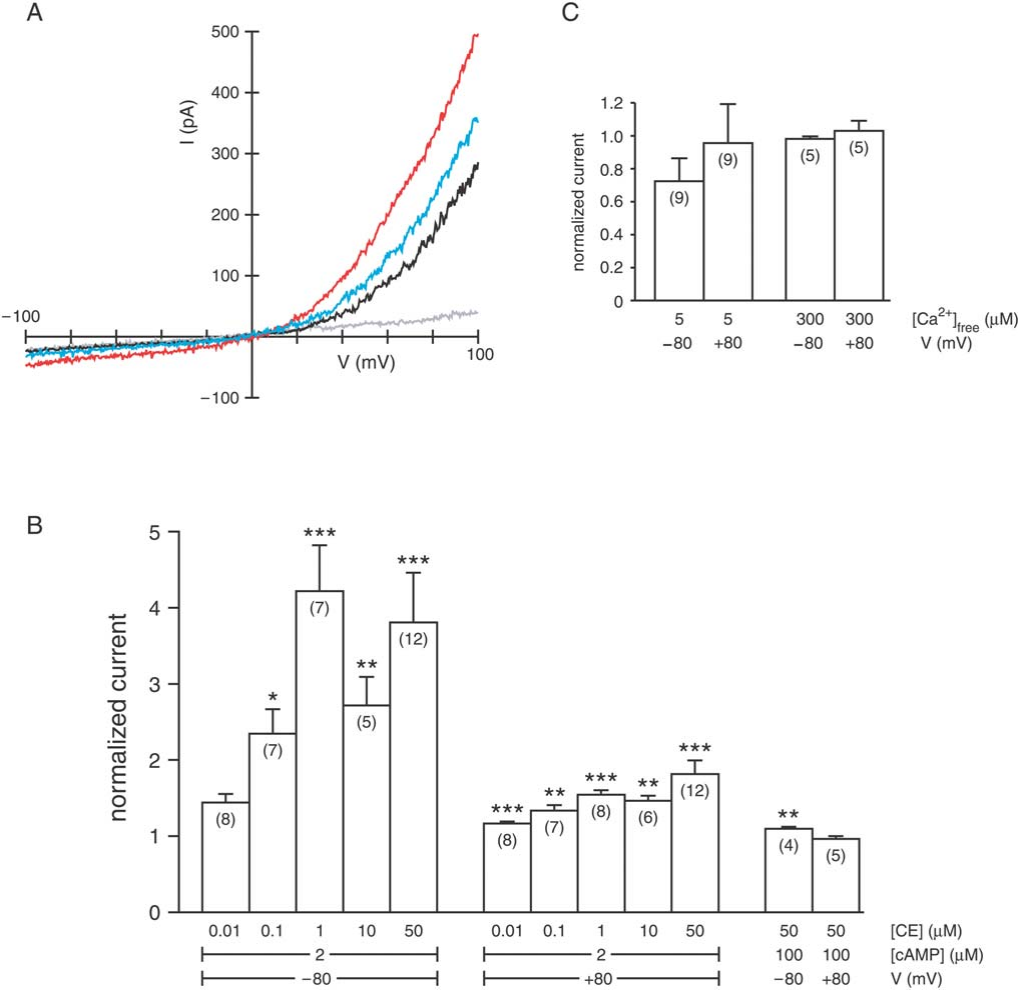
Effect of carboxyeosin (CE) on the ciliary transduction currents. (A) The current-voltage (I–V) relation of a cilium was measured with a 1-s voltage ramp in the following cytoplasmic baths: a control (gray) with no cAMP or CE; 2 µM cAMP (black); 2 µM cAMP and 50 µM CE (red); and 2 µM cAMP again (blue). Each bath also contained 0.1 µM free Ca^2+^, buffered with 2 mM BAPTA. Recordings were taken after 2 min in each bath, except that the last was taken after 4 min. (B) Summary of the effects of CE on the ciliary CNG current. Mean steady-state current was measured by averaging over a 2-s voltage step to −80 or +80 mV. Normalized current is the current measured in the presence of cAMP and CE divided by that measured in cAMP alone. The normalized currents shown are (left to right) 1.44±0.11, 2.35±0.32, 4.22±0.61, 2.72±0.37, 3.81±0.65, 1.17±0.03, 1.33±0.07, 1.54±0.06, 1.47±0.07, 1.82±0.18, 1.10±0.03, and 0.96±0.03. ***P<0.001; **P<0.02; *P<0.05. In 3 cilia tested, the current measured in 2 µM cAMP at +80 mV was less than the saturating current (measured in 100 µM cAMP) by a factor of 0.62±0.07. (C) Summary of the effects of CE on the ciliary Ca^2+^-activated Cl^−^ current. Normalized current is the current measured in the presence of Ca^2+^ and CE divided by that measured in Ca^2+^ alone. The normalized currents shown are (left to right) 0.72±0.14, 0.96±0.22, 0.98±0.01, and 1.03±0.06. In 7 cilia tested, the current measured in 5 µM Ca^2+^ at +80 mV was less than the saturating current (measured in 300 µM Ca^2+^) by a factor of 0.52±0.09.

Carboxyeosin (CE) is an inhibitor of PMCA [13], [16], [25]–[29] but affects other transporters as well [30]. In testing its specificity, I unexpectedly found that cytoplasmic CE significantly increases current through the ciliary CNG channels in physiological solutions (Fig. 3A,B). The effect was slowly reversible (Fig. 3A). The increase in current was seen at both negative and positive potentials in each of the 34 cilia tested. The increase was significant at CE concentrations as low as 0.1 µM (−80 mV) or 0.01 µM (+80 mV). It was substantial at a subsaturating concentration of cAMP (2 µM) but barely detectable with 100 µM cAMP (Fig. 3B). No significant effect of CE on the ciliary Ca^2+^-activated Cl^−^current could be found at either a half-maximal (5 µM) or saturating (300 µM) concentration of cytoplasmic free Ca^2+^ (Fig. 3C). To ensure that the effects of CE were not related to ATP-dependent transporters, ATP was not included in these bath solutions.

## Discussion

Numerous studies suggest that PMCA may contribute to the clearance of Ca^2+^ that accumulates within the olfactory cilia during the odor response [11]–[16]. Two questions can be considered in this regard: (1) how much does PMCA contribute to the Ca^2+^ clearance, and (2) is this PMCA activity located in the cilium?

Recent physiological studies of single olfactory receptor neurons have yielded results consistent with a role for PMCA in clearing Ca^2+^ following activation of the odor-transduction cascade [13]. In one case, caged cAMP was photolyzed within a primary olfactory neuron to produce a receptor current analogous to that caused by stimulation with odors [13]. Part of this current is carried by Ca^2+^. If PMCA contributes to clearance of this Ca^2+^, then carboxyeosin (CE), an inhibitor of PMCA [28], should prolong the elevation of cytoplasmic Ca^2+^. That in turn should prolong the Ca^2+^-activated Cl^−^ component of the transduction current. CE (50 µM) was found to prolong the transduction current. In a second study, a lower concentration of CE (10 µM) also prolonged the current but to a lesser extent [16]. These results are expected if CE inhibits PMCA.

However, a second mechanism may have contributed to this result. CE has a surprising effect on the CNG transduction channels; at concentrations of 0.1 µM or higher, it substantially increases the current activated by subsaturating concentrations of cAMP (Fig. 3A,B). Such an enhancement of current through the CNG channels could in part account for the prolonged transduction current measured in neurons treated with CE. The mechanism by which CE increases current through the CNG channels is unknown. In physiological solutions, the effect of CE is most effective at negative potentials (Fig. 3B), where external divalent cations reduce the CNG current by open-channel block (Fig. 3A). Over the full range of CE concentrations that typically block PMCA (2 to 50 µM; [refs. 13], [16], [25]–[29]), CE strongly enhances the CNG current. In the olfactory system at least, this makes CE an imperfect option for the study of PMCA. In the future, the proposed role for PMCA in Ca^2+^ clearance may be further supported by testing specific peptide inhibitors of PMCA [30] or PMCA-knockout animals.

It is also established that removal of cytoplasmic ATP prolongs the transduction current in olfactory receptor neurons [13]. Removal of ATP is predicted to shut down PMCA activity. As before, this should cause cytoplasmic Ca^2+^ to remain elevated, thus increasing the duration of the transduction current. Again, though, it is likely that a second mechanism contributes to this result. Na^+^/Ca^2+^ exchange contributes to the extrusion of cytoplasmic Ca^2+^ following the odor response [13], [31]–[34]. As cytoplasmic Ca^2+^ is extruded by exchange, cytoplasmic Na^+^ should accumulate within the cilium. It is possible that such an accumulation of Na^+^ might greatly reduce Na^+^/Ca^2+^ exchange unless the Na^+^,K^+^-ATPase can expel Na^+^ at a comparable rate [35]. Thus inhibition of Na^+^,K^+^-ATPase (as by elimination of ATP) might indirectly cause a shutdown of Na^+^/Ca^2+^ exchange, and this too could account for the dependence of Ca^2+^ clearance on ATP. A relation between Na^+^/Ca^2+^ exchange and Na^+^,K^+^-ATPase has been demonstrated in other systems [36] but not in olfactory cilia. Na^+^,K^+^-ATPase is present in olfactory cilia [37], [38]. This mechanism could also contribute to the effect of CE on the olfactory transduction current. Eosin, from which CE is derived, inhibits all ATPases [30]. At some concentration, CE may inhibit the Na^+^,K^+^-ATPase. At present, the studies with CE and ATP [13] are the only physiological evidence linking PMCA to the kinetics of the olfactory transduction current.

A second question involves the cellular location of the mechanisms mediating clearance of Ca^2+^ from the cilium. Are the Ca^2+^ transporters located in the cilium itself? In models of the cilium, Ca^2+^ clearance is insufficient unless a Ca^2+^ transporter is assumed to operate in the ciliary membrane [35], [39]. Physiological evidence indicates that Na^+^/Ca^2+^ exchange occurs in the cilium [33]. A ciliary location for PMCA is also supported by immunological studies in toad [13], rat [13], and mouse [12] and proteomic studies in rat [14], [15]. PMCA activity has been demonstrated in membrane vesicles derived from olfactory cilia [11], [13]. Some Ca^2+^ may be cleared by diffusion into other cellular compartments. However, it appears that the free Ca^2+^ produced during a moderate odor response may extend just ∼2 µm from the site of odor binding [40]. It is likely that cytoplasmic Ca^2+^ buffers also help to limit free Ca^2+^. In some cellular compartments, Ca^2+^ can also be sequestered within endoplasmic reticulum or mitochondria [1]. However, ultrastructural descriptions of olfactory cilia have not reported the presence of endoplasmic reticulum or mitochondria [2], [41]–[43]. Thus it is generally believed that the cilia lack these organelles.

Estimates of the intraciliary free Ca^2+^ concentration during the odor response range from 300 nM [8] to 100 µM [9]. Cl^−^ channels are gated during a moderate response [5], [7], [33]. Given the dose-response relation for these channels [19]–[21], cytoplasmic Ca^2+^ must exceed ∼2 µM during such a response, at least in local domains. In ciliary membrane vesicles, PMCA is half-maximally and maximally effective at free Ca^2+^ concentrations of 0.67 and 5 µM, respectively [13]. One would thus predict that PMCA should actively expel the micromolar concentrations of free Ca^2+^ seen during the odor response. While examining cilia in isolation, though, I was unable to detect ATP-dependent expulsion of Ca^2+^ over the range of Ca^2+^ concentrations detectable by the assays (2 to 10 µM). The second assay (Fig. 2) closely mimics the normal entry of Ca^2+^ into the cilium during the odor response. Recording from a single cilium has the advantage of eliminating transport activities in other cellular compartments. At the same time, it must be acknowledged that ciliary excision could allow the loss of cytoplasmic factors needed for normal physiological function.

The results of the present study do not obviously conflict with previous reports. In the cilium, PMCA and Na^+^/Ca^2+^ exchange may both be active at the levels of free Ca^2+^ generated during the odor response. However, due to its much lower capacity [1], [10], PMCA may account for only a small fraction of the Ca^2+^ expulsion. In this case, the rate of Ca^2+^ expulsion by PMCA could be too small to be detected by the assays used here. PMCA in nonciliary compartments, which were not studied here, may also contribute to Ca^2+^ clearance.
